# A Spectroscopic Determination of Scattering Lengths for Sodium Atom Collisions

**DOI:** 10.6028/jres.101.051

**Published:** 1996

**Authors:** Eite Tiesinga, Carl J. Williams, Paul S. Julienne, Kevin M. Jones, Paul D. Lett, William D. Phillips

**Affiliations:** National Institute of Standards and Technology, Gaithersburg, MD 20899-0001

**Keywords:** laser cooling, photoassociation spectroscopy, scattering length, spectral line shapes, ultracold sodium atom collisions

## Abstract

We report a preliminary value for the zero magnetic field Na ^2^S(*f* = 1, *m* = − 1) + Na ^2^S(*f* = 1, *m* = − 1) scattering length, *a*_1,−1_. This parameter describes the low-energy elastic two-body processes in a dilute gas of composite bosons and determines, to a large extent, the macroscopic wavefunction of a Bose condensate in a trap. Our scattering length is obtained from photoassociative spectroscopy with samples of uncondensed atoms. The temperature of the atoms is sufficiently low that contributions from the three lowest partial waves dominate the spectrum. The observed lineshapes for the purely long-range $0_{g}^{-}$ molecular state enable us to establish key features of the ground state scattering wavefunction. The fortuitous occurrence of a *p*-wave node near the deepest point (*R*_e_ = 72 *a*_0_) of the $0_{g}^{-}$ potential curve is instrumental in determining *a*_1,−1_ = (52 ± 5) *a*_0_ and *a*_2,2_ = (85 ± 3) *a*_0_, where the latter is for a collision of two Na ^2^S(*f* = 2, *m* = 2) atoms.

## 1. Introduction

Last year two groups reported the observation of Bose-Einstein Condensation (BEC) in dilute gasses of ultra-cold ^87^Rb and ^23^Na [1,2], and another reported evidence for reaching the quantum degenerate regime in ^7^Li [3] but without observing BEC [4]. The observation of BEC in a weakly-interacting gas opens up a whole range of possibilities, from fundamental studies of the coherent atomic samples produced, to the construction of the atom-analog of a laser. Theoretical descriptions of the weakly interacting Bose condensate are only now being developed and experimental techniques to probe the condensate are just beginning to be explored.

One of the fundamental parameters required to understand the approach to BEC and the properties of the condensate is the *s*-wave scattering length. This scattering length determines the low energy elastic scattering rate and thus the evaporative cooling rate as well as the nonlinear coupling parameter in the Gross-Pitaevski equation [5] for the condensate wavefunction. It is not necessary to produce a condensate to measure the *s*-wave scattering length: temperatures in a magneto-optic trap (MOT) are sufficiently low (≈ 1 mK) to limit scattering to a few partial waves and thus permit a determination of the *s*-wave scattering length.

We probe the scattering wavefunction using the technique of photoassociation spectroscopy [6–10]. Two Na atoms colliding along the ground state 3^2^S + 3^2^S potential can absorb a photon to produce a bound molecule, in our case to vibrational levels with energy near the 3^2^S + 3^2^P_3/2_ asymptote. We detect the formation of molecules by sending in a second photon which excites the molecule to an autoionizing state, thereby producing an easily detected ${\mathrm{Na}}_{2}^{+}$ ion. The relative intensities of the molecular photoassociation lines carry information about the ground state wavefunction. In particular, we find that two specific rovibrational lines that arise from *p*-wave scattering are significantly weaker than the corresponding lines for other nearby vibrational levels. This indicates that the former rovibrational state is centered at an internuclear separation near a node in the *p*-wave ground state wavefunction. With the location of this node established, the intensities and lineshapes of other rovibrational lines allow us to constrain the location of the corresponding *s*-wave node, and thus to determine the scattering length.

**Fig. 1:**
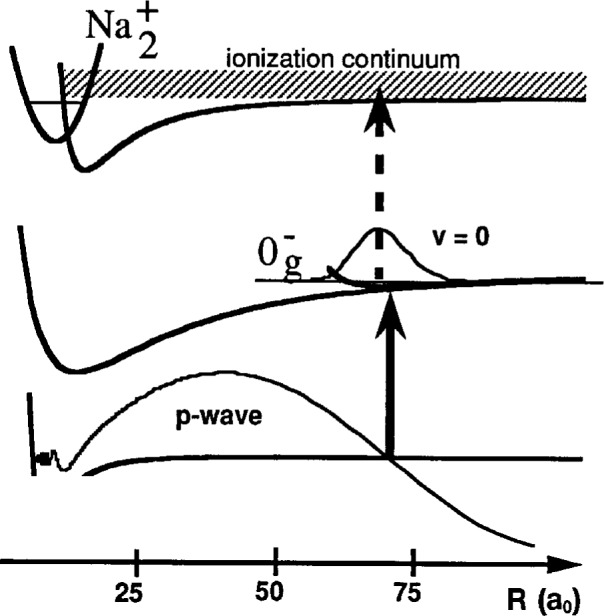
Sketch of the two-step photoassociation/molecular ionization process used to obtain the spectrum of the $0_{g}^{-}$ state. Two colliding atoms approach along the ground state molecular potential and are excited to a bound molecular state by a laser photon (solid arrow). The excited molecules thus created are then excited to an autoionizing continuum by the second laser (dashed arrow). The *p*-wave ground state scattering wavefunction is shown with a node directly underneath the *R*_e_ of the $0_{g}^{-}$ potential. This leads to an absence of *p*-wave features (odd rotational lines) in the experimental spectrum of the *v* = 0 vibrational level.

The transitions which we use are from two colliding Na 3^2^S(*f* = 1) atoms to the ${\mathrm{Na}}_{2}0_{g}^{-}$ “purely long range” molecular state which asymptotically correlates to a 3^2^S and a 3^2^P_3/2_ atom [11–15]. The wavefunctions of the lowest vibrational levels in this potential are localized at distances between 50 *a*_0_ and 100 *a*_0_, as shown in Fig. 1. (The Bohr radius *a*_0_ = 0.0529177 nm.) This molecular potential is determined almost entirely by the known long range forces between atoms and the magnitude of the atomic spin-orbit splitting, and thus may be calculated to high precision. The transition rate depends on the overlap between the ground state wavefunction for a low energy collision and the excited bound state wavefunction. It is a fortuitous coincidence that there is a node in the *p*-wave scattering wavefunction that is nearly centered on the minimum of the $0_{g}^{-}$ potential. This leads to an almost complete cancellation of the overlap integral between the Na ^2^S(*f* = 1, *m* = − 1) + Na ^2^S(*f* = 1, *m* = − 1) *p*-wave scattering wavefunction and the symmetric *v* = 0 vibrational wavefunction, resulting in a striking and characteristic absence of *p*-wave features in the spectrum of the *v* = 0 level of the $0_{g}^{-}$ state in our experiments. We are able to construct a family of ground state potentials consistent with the known spectroscopy of the molecular ground states that also reproduce the *p*-wave node near the minimum of the $0_{g}^{-}$ state. We obtain further constraints on the acceptable potentials from the width and the relative heights of the rotational features in the spectrum. This, in turn, places constraints on the position of the corresponding *s*-wave node. Finally, we relate the *s*-wave nodal position to the scattering length.

## 2. Experimental Spectra and Lineshapes

The experiments are performed by loading Na atoms into a “dark spot” MOT [16]. The trapping lasers are turned off for brief periods (~ 10 μs) and a tunable probe laser is introduced during this time. For selected frequencies of the probe laser, red of the atomic resonance, pairs of atoms undergoing collisions are excited to molecular states. These molecules are then detected by ionization with a second probe laser. The ionization laser is tuned to be non-resonant with any photoassociating transition but to allow ionization of the molecular states of interest. Measurements such as these have been described before [8,15], and here we review only those features important for the understanding of the analysis below.

The MOT captures Na atoms using the 3^2^S(*f* = 2) → 3^2^P_3/2_(*f* = 3) atomic transition. This transition is not a closed cycling transition because occasionally atoms get excited to the 3^2^P_3/2_(*f* = 2) state which can decay to the 3^2^S(*f* = 1) state, requiring the “repumping” of atoms that fall into the 3^2^S(*f* = 1) ground state. The dark spot MOT has this repumping frequency missing from the central volume of the trap and, consequently, the atoms are almost completely optically pumped into the 3^2^S(*f* = 1) ground state. All of the transitions we discuss in this paper begin from the 3^2^S(*f* = 1) + 3^2^S(*f* = 1) ground state. When the photoassociating probe is introduced there are no excited state atoms present. The ionizing laser present during the probe periods is tuned blue of the atomic resonance frequency and does not affect the atoms in the MOT. The ionizing laser frequency is chosen and kept fixed while the photoassociating laser is scanned over the ≈ 1 GHz frequency range spanned by the rotational structure of a given $0_{g}^{-}$ vibrational level. We check that the laser powers are low enough that the signal heights are linear and that the linewidths are independent of power.

The frequency of the ionizing laser is chosen to take the molecules formed in the photoassociation step into the ionization continuum (see Fig. 1) just above the 3^2^P_3/2_ + 3^2^P_3/2_ asymptote. This continuum has structure [8] which complicates the interpretation of the spectra presented here. If the sum of the two laser frequencies (photoassociating plus ionizing) coincides with a narrow feature in the continuum for some particular frequency range of the photoassociating laser then the relative intensities of the rotational lines will not be proportional to the transition strengths in the photoassociation step. Since these relative transition strengths are important for our analysis, we work in a region where there are no sharp resonances and the ionization continuum is not rapidly varying. Nonetheless, this does lead to some uncertainty in the relative intensities of the experimental peaks.

**Fig. 2:**
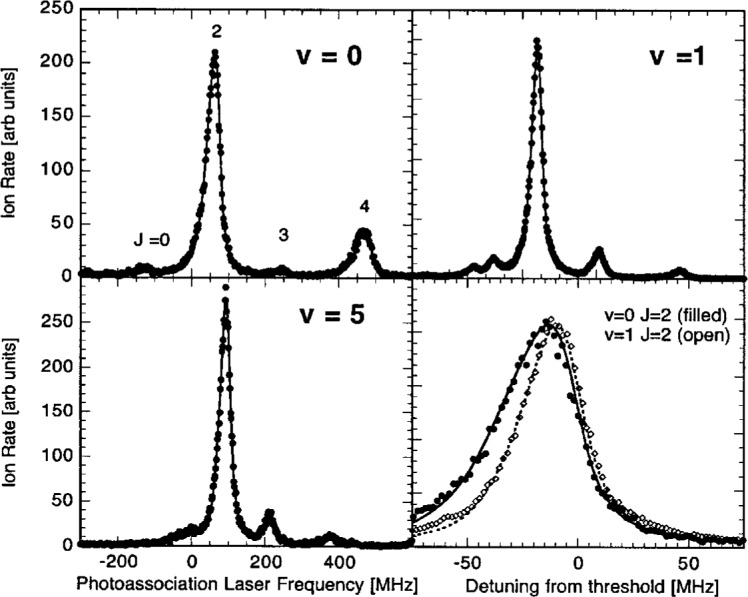
Experimental rotational progressions for the *v* = 0, 1, and 5 vibrational levels of the $0_{g}^{-}$ state. Each panel spans 900 MHz except for the lower right which is an expanded comparison of the *v* = 0 and *v* = 1, *J* = 2 peaks, showing that the *v* = 0 feature has a larger width. The fitted curves are *s*-wave [see Eq. (2)] for *J* = 0 and 2, *p*-wave for *J* = 1 and 3, and *d*-wave for *J* = 4, except for the *v* = 0, *J* = 2 peak for which there is a strong *d*-wave contribution. The temperature is fixed at *k*_B_*T*/*h* = 9 MHz and the natural line width is set to 20 MHz for *v* = 0 and 22 MHz for *v* = 1 and 5 (to allow for unresolved hyperfine structure).

Figure 2 shows spectra of several $0_{g}^{-}$ vibrational levels. Several observations can immediately be made. The spectra show a rotational progression of lines at positions given by *B_v_J*′(*J*′ + 1), where only the lowest five *J*′ features are visible (*J*′ = 0 – 4), and *B_v_* is the rotational constant for vibrational level *v*. The *J*′ = 2 peak is always much larger than the other rotational lines. For the *v* = 0 vibrational level the odd *J*′s are nearly absent, while for *v* = 1 these odd *J*′ peaks are clearly visible. In fact the odd *J*′ peaks are larger than the *J*′ = 0 and 4 lines. The *v* = 5 spectrum is typical for the *v* > 2 levels. Moreover, for *v* = 0 the ratio of the heights of the *J*′ = 4 and the *J*′ = 2 peaks is of the order of 0.2. Changing the frequency of the ionizing laser can change this ratio by approximately a factor of two. Finally, for all the vibrational levels examined up to *v* = 8 the *J*′ = 2 peak, with a width of ≈ 30 MHz, is narrower than the *J*′ = 4 peak and is more symmetric as well.

The observed lineshapes are understood as a Lorentzian profile convolved with the thermal distribution of the ground state collision energies [6]. The lineshape for a given vibrational-rotational level (*v*, *J*′) is proportional to the following lineshape factor:

$$\begin{array}{c} S\left(\omega ,T,v,J\prime \right)=\sum_{F\prime p\prime \beta ,Fp\ell f\alpha }n_{\alpha }\left(2F\prime +1\right)\\ \int_{0}^{\infty }dEe^{-E/k_{B}T}\frac{\gamma_{o}|\langle \phi_{F\prime p^{\prime }\beta }^{vJ\prime }|\hbar \Omega_{Fp\ell f\alpha }^{F\prime p\prime \beta }|\Psi_{Fp\ell f\alpha }^{E\left(+\right)}\rangle {|}^{2}}{{\left(E+\hbar \omega -E_{F\prime p\prime \beta }^{vJ\prime }\right)}^{2}+{\left(\gamma_{v}/2\right)}^{2}} \end{array}$$ (1)

where *ω* is the laser frequency, *T* is the temperature of the sample, $E_{F\prime p\prime \beta }^{vJ\prime }$, $|\phi_{F\prime p\prime \beta }^{vJ\prime }\rangle$, and *γ_v_* are the excited state energy, wavefunction, and natural linewidth respectively. The excited state wavefunction is labeled by the total angular momentum quantum number *F*′, the parity *p*′ and the remaining hyperfine and electronic degrees of freedom labeled *β*. In addition, it is labeled with the vibrational quantum number *v* and rotational quantum number *J*′, where ***J*′** = ***F*′** − ***I*** and *I* is the total nuclear spin angular momentum quantum number [13]. The summation over *F′p′β* in Eq. (1) for a (*v*,*J*′) level is due to the (unresolved) hyperfine structure of the $0_{g}^{-}$ state. The ground collisional wavefunction represented by $|\Psi_{Fp\ell f\alpha }^{E\left(+\right)}\rangle$ is energy normalized, the subscripts denote the spin channel |*Fp*ℓ*fα*〉 in which the collision starts, and the + indicates the proper scattering boundary conditions [17]. *F* is the ground state total angular momentum, *p* is the parity, and *E* is the asymptotic kinetic energy. The total angular momentum of the system can be written as ***F*** = **ℓ** + ***f***_a_ + ***f***_b_ = **ℓ** + ***f***, where ***f***_a_ and ***f***_b_ are the asymptotic total angular momenta of the two atoms, **ℓ** is the mechanical rotation, ***f***—the vector sum of ***f***_a_ and ***f***_b_—is a generalized spin label, and *α* uniquely labels the remaining degrees of freedom of the asymptotic atomic scattering states for the 3^2^S(*f*_a_ = 1) + 3^2^S(*f*_b_ = 1) collision. The quantity *n_α_* is the population of the collision channel labeled by *α*. To avoid confusion between the atomic and molecular labels we will hereafter label individual atomic hyperfine states by *f*_a_ or *f*_b_ while *f* will be used solely to denote the vector sum of *f*_a_ and *f*_b_. Finally, $\Omega_{Fp\ell f\alpha }^{F\prime p\prime \beta }$ is the electronic optical transition matrix element between the ground state labeled by *Fp*ℓ*fa* and the excited state labeled by *F′p′b*. The rate *γ*_o_/ℏ is the rate at which the excited *vJ*′ level produces observable products, in this case, the photoionization rate by the second laser. Here the photoionization contributes negligibly to the total width: *γ*_o_ ≪ *γ_v_*.

We assume that the absorption of the second photon does not modify the shape of the spectra. From changing the color of the second photon we have seen that this is not always a valid assumption. Nevertheless, the measurements indicate that, over a large range of frequencies of the second laser, the relative intensities of the main features that we are concerned with in the spectra are insensitive to this.

For ultracold atom-atom collisions the matrix element of the dipole moment has a kinetic energy dependence governed by the Wigner-threshold law [18,19], that is, the initial collision wavefunction $|\Psi_{Fp\ell f\alpha }^{E\left(+\right)}\rangle$ is proportional to *E*^(2*ℓ*+1)/4^. For example, for *s*-wave scattering the wavefunction is proportional to $\sqrt[{4}]{E}$. Due to this Wigner-law variation in the (Franck-Condon) matrix element, Eq. (1) leads to asymmetric lineshapes [6] where the blue side is dominated by the Lorentzian in Eq. (1) and the red side is predominantly determined by the Maxwell-Boltzmann distribution of kinetic energies. The observed position of the peak is always red shifted with respect to the actual bound state energy *E_vJ′_*. This shift is on the order of *k*_B_*T*, the linewidth is on the order of *k*_B_*T* + *γ_v_*, and both increase with *ℓ*.

For each *J*′ we fit the line to

$$\begin{array}{c} S_{\text{fit}}\left(\omega ,T,v,J\prime \right)=A_{vJ\prime }\\ \int_{0}^{\infty }dEe^{-E/k_{B}T}\frac{E^{\left(2\ell +1\right)/2}}{{\left(E+\hbar \omega -E_{v,J\prime }\right)}^{2}+{\left(\gamma_{v}/2\right)}^{2}}. \end{array}$$ (2)

The coefficient *A_vJ′_* is the overall amplitude, *E_v,J′_* is the transition threshold energy, *γ_v_* is the linewidth and *T* the temperature. The results of our fits are shown in Fig. 2. We use a single value of *T* for all of the data, determined from the fits to be (450 ± 50) μK (*k*_B_*T*/*h* = 9 MHz). For reasons discussed below, we fit the odd *J*′ features to Eq. (2) with ℓ = 1 (*p*-wave) only. The *J*′ = 0 and 2 peaks are fit to ℓ = 0 (*s*-wave), except for *v* = 0 where we find it necessary to use a sum of ℓ = 0 and ℓ = 2 contributions. The *J*′ = 4 peak is fitted with just ℓ = 2 (*d*-wave). The natural linewidth of the $0_{g}^{-}$ states is 20 MHz, which is twice the atomic linewidth [20,21]. For *v* = 0 we expect the unresolved hyperfine structure to broaden the line by ≈ 2 MHz. To fit the *v* = 0, *J*′ = 2 peak with a single *s*-wave lineshape requires an unrealistically large (30 MHz) linewidth, whereas for *v* = 1, where the hyperfine splitting is slightly larger, a linewidth of only 22 MHz is required to fit the data. We return to these points in Secs. 3 and 4.

## 3. General Theory

The theory which underlies our calculation of the spectrum involves three major pieces: the ground state wavefunctions, the excited state wavefunctions and the molecular Rabi matrix which gives the optical coupling between them. These determine the transition amplitude matrix element $\langle \phi_{F\prime p\prime \beta }^{vJ\prime }|\hbar \Omega_{Fp\ell f\alpha }^{F\prime p\prime \beta }|\Psi_{Fp\ell f\alpha }^{E(+)}\rangle$, from which we calculate synthetic spectra to compare to experiment.

The first piece is the ground state wavefunction $|\Psi_{Fp\ell f\alpha }^{E\left(+\right)}\rangle$, which is obtained from an exact solution of the Schrödinger equation for the ground state Hamiltonian $H_{\text{ground}}^{Fp}$ for a given set of adiabatic Born-Oppenheimer (ABO) potentials which are derived from experimental Rydberg-Klein-Rees (RKR) potentials. The ground state Hamiltonian $H_{\text{ground}}^{Fp}$ is set up for a given value of the total angular momentum and parity and includes electrostatic interactions *V*(*R*) (the adiabatic Born-Oppenheimer potentials), the mechanical rotation operator ${\hat{\ell }}^{2}/2\mu R^{2}$, the radial kinetic energy operator, the spin-spin dipole interaction, and the atomic hyperfine Hamiltonians. Most of our discussion will use a simpler model of $H_{\text{ground}}^{Fp}$ and $|\Psi_{Fpf\ell \alpha }^{E\left(+\right)}\rangle$ since this provides greatly improved insight. We note that although the discussions may be based upon simpler, intuitive models the final calculations use the full $H_{\text{ground}}^{Fp}$ and $|\Psi_{Fp\ell f\alpha }^{E\left(+\right)}\rangle$.

The next piece of the theory required to model the photoassociation spectra is to calculate the excited rovibrational-hyperfine wavefunctions $|\phi_{F\prime p\prime \beta }^{vJ\prime }\rangle$ and energies $E_{F\prime p\prime \beta }^{vJ\prime }$. Once again, these are obtained from an exact treatment of the excited state Hamiltonian $H_{\text{excited}}^{F\prime p\prime }$ which includes the same interactions for the excited state as were contained in $H_{\text{ground}}^{F,p}$ plus a spin-orbit interaction that results from the presence of the excited Na 3^2^P atom, and retardation of the excited resonance dipole interaction. A discussion of $H_{\text{excited}}^{F\prime p\prime }$ and methods for finding its bound state solutions are found in Refs. [13] and [14]. Once again, most of our discussion will be based on a simple one channel adiabatic picture of the $0_{g}^{-}$ bound states although the exact bound state wavefunctions and energies are used in the calculations.

Finally, we need the molecular Rabi matrix elements $\Omega_{Fp\ell f\alpha }^{F\prime p\prime \beta }$ between the initial ground electronic state labeled by ℓ*fα* and the excited electronic state labeled by *β*. Dipole selection rules require that *p*′ = − *p*, and Δ*F* = *F*′ − *F* = {0, ± 1}, except that Δ*F* ≠ 0 for *F* = 0. The $\Omega_{Fp\ell f\alpha }^{F\prime p\prime \beta }$ are calculated from the known atomic transition dipole moment between a ground Na 3^2^S atom and an excited 3^2^P atom using the basic approach described in Ref. [21] but generalized here to include hyperfine structure. The molecular Rabi matrix elements depend on the excited rovibrational-hyperfine state quantum numbers, *F′p′βvJ*′, and the ground state hyperfine levels *f*_a_ and *f*_b_ of the two colliding atoms.

These three pieces of theory are integrated together using Eq. (1) to yield a theoretical spectra which can be compared to the experimental spectra. We know that we can calculate the excited state $0_{g}^{-}$ bound state energies to an accuracy of a few MHz [13] and have used this capability to determine a precision value of the Na 3^2^P_3/2_ lifetime and to provide the first experimental verification of retardation of the interaction between two atoms [14].

Below we will briefly describe each of these three theoretical parts while emphasizing those portions relevant to the current problem of extracting ground state scattering lengths. Many arguments will take advantage of simple physical pictures. These pictures are meant to be intuitive and they have been verified within the context of two colliding Na atoms where possible. However, we note that the final results are based on the full Hamiltonian, the exactly calculated ground and excited state wavefunctions, and the hyperfine labeled electronic transition dipole moment between the initial and final hyperfine labeled electronic states.

### 3.1 Ground State Dynamics

Although we have set up a complete quantum scattering calculation for two ground state atoms with hyperfine structure, as described in the previous section, a sufficiently accurate model of ^2^S + ^2^S collisions is obtained with the atomic hyperfine Hamiltonian for each atom, the ground $X^{1}\sum_{g}^{+}$ and $a^{3}\sum_{u}^{+}$ molecular potentials, the mechanical rotational kinetic energy, and the − *ℏ*^2^/2*μ* · d^2^/d*R*^2^ radial kinetic energy (where the reduced mass *μ* equals half the atomic ^23^Na mass). This approximate model ignores the very weak magnetic spin-spin interactions and the second-order spin-orbit interaction with distant electronic states. In the absence of these weak spin-dependent terms in the Hamiltonian, the mechanical rotation ℓ is a conserved quantum number. This does not imply that ℓ -changing collisions are always irrelevant. In fact, in experiments aiming at Bose condensation, atom loss is in a large part due to such processes, which can always be treated using a weak interaction picture [22,23]. However, spin interactions play a negligible role in the description of the spectra obtained with photoassociative spectroscopy.

The electrostatic $X^{1}\sum_{g}^{+}$ and $a^{3}\sum_{u}^{+}$ potentials over part of the range of their attractive wells have been derived from conventional spectroscopy [24]. We extrapolate these RKR potentials by joining them smoothly to the familiar long-range dispersion form $V_{\text{disp}}=-\sum_{n=6}^{\infty }C_{n}/R^{n}$ using the coefficients of Ref. [25]. Note that for *R* > 30 *a*_0_ these two adiabatic Born-Oppenheimer potentials are essentially identical and are, at 30 *a*_0_, about *V*_disp_/*k*_B_ = − 0.7 K deep. These potentials predict that the $X^{1}\sum_{g}^{+}$ state has 65 *s*-wave vibrational levels while the $a^{3}\sum_{u}^{+}$ potential has 15 *s*-wave levels [24,26]. The scattering length associated with each potential is sensitive to the precise phase of the wavefunction at zero energy, which is related to the binding energy of the last bound state. Uncertainty in the extrapolation of the RKR region of the potential leads to uncertainty in the exact position of the last ground state vibrational level, and consequently uncertainty in the scattering length. It is the sensitivity of the photoassociation spectra to the phase of the low energy ground state wavefunction (i.e., to the position of the nodes in the wavefunction) that allows us to obtain the scattering lengths associated with the collision of particular hyperfine states. In order to reproduce the experimental $0_{g}^{-}$ lineshapes we will allow the shape of the inner wall of the electrostatic $X^{1}\sum_{g}^{+}$ and $a^{3}\sum_{u}^{+}$ potentials to vary in order to adjust for short and long range extrapolation uncertainties, but we restrict the changes to conserve the number of levels in these two potentials. In practice, the inner walls of the two RKR curves are allowed to vary independently.

In the dark spot MOT the sodium atoms are in the atomic *f*_a_ = 1 hyperfine state and are assumed to be distributed equally over the three magnetic sublevels $m_{f_{a}}$. Since the MOT has a nearly-zero magnetic field (< 0.1 mT and spatially-varying in magnitude and direction), collisions are independent of the orientation of the molecule in the laboratory frame. We may view the collision as starting when the atoms are infinitely far apart with a definite value for the relative angular momentum ℓ and retaining this value throughout the collision. We can therefore evaluate the ground state Hamiltonian in the atomic hyperfine basis |*Fp*ℓ*fα*〉 for fixed values of the total angular momentum ***F*** = **ℓ** + ***f*** and parity *p*, where here *α* designates {*f*_a_*f*_b_}. The parity *p* is the symmetry of the ^2^S + ^2^S Hamiltonian under inversion through the center of mass of all the electron and nuclear coordinates. Since the angular momentum **ℓ** is conserved during the collision, coupling to ***F*** is not really necessary but is useful in setting up the molecular Rabi matrix below. The rotational and hyperfine Hamiltonian terms are diagonal in this atomic hyperfine basis, although the electrostatic terms are not, since the basis does not form states with good electron spin ***S*** = ***s***_a_ + ***s***_b_. However, when we neglect the weak spin-spin coupling terms, there is a diagonal representation in a molecular basis with *S* and ℓ as good quantum numbers:

$$|Fp\ell fSI\rangle \propto \sum_{f_{a}f_{b}}\sqrt{\left(2S+1\right)\left(2I+1\right)\left(2f_{a}+1\right)\left(2f_{b}+1\right)}\times \left\{\begin{array}{ccc} s_{a} & i_{a} & f_{a}\\ s_{b} & i_{b} & f_{b}\\ S & I & f \end{array}\right\}|Fp\ell ff_{a}f_{b}\rangle$$ (3)

where {…} is a nine-*j* symbol; the exact equation has phase and normalization factors resulting from nuclear symmetrization. Since the Born-Oppenheimer curves do not depend on *f* it is a conserved quantity. There is also a restriction on the permissible quantum numbers due to the homonuclear nature of the dimer since the basis states must be antisymmetric with respect to exchange of the two nuclei. This leads to the restriction (− 1)^ℓ+^*^σ^*^+^*^I^* = 1 with *σ* = 0(1) for gerade (ungerade) states (for ^2^S + ^2^S collisions there also exists a one-to-one correspondence between gerade/ungerade and the total electron spin *S*, allowing *S* to be substituted for *σ*). In the atomic basis the restriction is ${\left(-1\right)}^{\ell +f-f_{a}-f_{b}}=1$. An important consequence is that the Na^2^S(*f*_a_ = 1) + Na^2^S(*f*_b_ = 1) spin state couples to even *f* = 0 or 2 for even partial waves and to odd *f* = 1 for odd ℓ’s. This latter statement is true whether or not we neglect the weak spin-spin interactions.

The fact that ℓ and *f* are good approximate quantum numbers lets us develop a relatively simple picture of photoassociation spectra due to collisions of ^2^S(*f*_a_ = 1) + ^2^S(*f*_b_ = 1) atoms. There are only two possible *s*-wave contributions, corresponding to *f* = 0 and *f* = 2. These have *F* = 0 and *F* = 2 respectively. For the *p*-wave there is only one possible contribution, corresponding to *f* = 1 and *F* = 0, 1, or 2. Finally, there are two possible *d*-wave contributions, where *F* = 2 for *f* = 0 and *F* = 0, 1, 2, 3, or 4 for *f* = 2. Within our approximation of neglecting weak spin-spin interactions a given *f*, ℓ subspace contained in Hamiltonians labeled by different *F*’s are identical, with identical wavefunctions. Thus, the three values of *F* which contain the *f* = 1, ℓ = 1 subspace have identical *p*-waves and thus identical nodes. Therefore, we can represent the collision in terms of two *s*-waves, one *p*-wave, and two *d*-waves. For brevity we will refer to these five wavefunctions as $\Psi_{\ell f}^{\left(+\right)}$ and thus as $\Psi_{s0}^{\left(+\right)}$, $\Psi_{s2}^{\left(+\right)}$, $\Psi_{p1}^{\left(+\right)}$, $\Psi_{d0}^{\left(+\right)}$ and $\Psi_{d2}^{\left(+\right)}$.

BEC experiments can magnetically trap the alkali-metal atoms in one of the magnetic sublevels. There are two relevant states. One is the doubly polarized state where all atoms are in the atomic *f*_a_ = 2 and $m_{f_{a}}=2$ state. Two of these atoms have a projection of $m_{f}=m_{f_{a}}+m_{f_{b}}=4$ which implies *f* = 4. The second trappable spin state, used by the MIT group [2], is the *f*_a_ = 1 and $m_{f_{a}}=-1$ state. This implies that a collision between two such states couple to a |(*f*_a_ = 1,*f*_b_ = 1)*f* = 2, *m_f_* = −2〉 state. The zero-field scattering length of the latter state is extracted from our experiment; in fact, it is related to $\Psi_{s2}^{\left(+\right)}$. Because the magnetic fields used in the sodium traps of Ref. [2] are weak, the Zeeman shifts of the atomic hyperfine states are small compared to the hyperfine structure and thus have little effect on the collision dynamics. Hence the zero-field scattering length is the relevant parameter in those experiments.

**Fig. 3:**
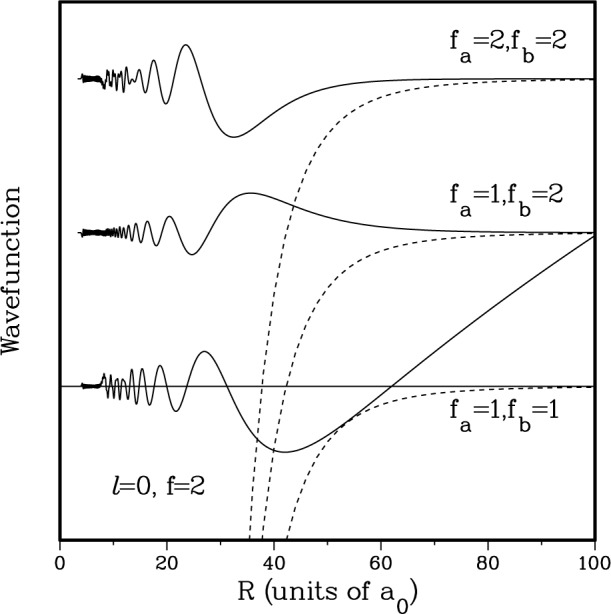
The multichannel S + S collisional wavefunction as a function of internuclear separation. The wavefunction describes an *E*/*k*_B_ = 500 μK *s*-wave collision of two *f_α_* = 1 atoms coupled to a *f* = *f*_a_ + *f*_b_ = 2 state. Two of the three spin components *ψ* of the wavefunction decay exponentially because those states are asymptotically unaccessible. The dashed lines denote the attractive long-range dispersion potential for each of the three spin channels. The horizontal line denotes the total energy in the collision.

The ^2^S + ^2^S collisional wavefunction is inherently multichannel. In Fig. 3 we show the three components of an exact close-coupling wavefunction [22,23,27], for an incoming *s*-wave in the *f*_a_ = 1, *f*_b_ = 1 channel with *f* = *F* = 2 and a kinetic energy of *E*/*k*_B_ = 500 μK. The figure also shows the three potential curves (dashed lines) for each of the three spin channels. The horizontal line indicates the total collision energy. The plane wave scatters into the two other *s*-waves with *f* = *F* = 2; they have *f*_a_ = 1, *f*_b_ = 2 and *f*_a_ = 2, *f*_b_ = 2 respectively. These other channels are closed asymptotically by *E*/*k*_B_ = + 85 mK and + 170 mK, respectively. Therefore, they are only populated at short internuclear separation, where the attractive potential is larger than the asymptotic separation and where the electrostatic exchange interaction (the difference between the *X*^1^Σ_g_ and *a*^3^Σ_u_ potentials) can mix these three spin channels. The mixing occurs around 25 *a*_0_, where the exchange splitting is comparable to the hyperfine splitting. Inside 20 *a*_0_ the wavefunction oscillates rapidly due to the high kinetic energy in the deep potentials and shows striking interference patterns due to the strong electrostatic interaction. In this region the “molecular” basis would be more appropriate than the atomic hyperfine one. For *R* > 30 *a*_0_ the three channels are decoupled and the dynamics is governed by the common long-range potential and the kinetic energy. The wavefunction components for the upper two channels decay to zero since these channels are closed, while the *s*-wave in the *f*_a_ = 1 + *f*_b_ = 1 entrance channel extends to *R* = ∞ with long wavelength oscillations. At large *R* this low-energy wavefunction (except for an *R* independent phase factor) is given by

$$\begin{array}{c} |\Psi_{Fp\ell ff_{a}f_{b}}^{E\left(+\right)}\rangle =\sum_{f_{a}f_{b}}\phi_{f_{a}f_{b}}^{\left(+\right)}\left(R\right)|F=2,p=+1,\ell =0,f=2\left(f_{a},f_{b}\right)\rangle \\ \to \sqrt{\frac{2\mu }{\hbar^{2}\pi }}\frac{1}{\sqrt{k}}\sin\left(k\left(R-a_{1,-1}\right)\right)\\ |F=2p=+1,\ell =0,f=2\left(f_{a}=1,f_{b}=1\right)\rangle ,\;R\to \infty \end{array}$$ (4)

with *k* the asymptotic wavenumber and *a*_1,21_ the scattering length.

Most notable about the wavefunction in Fig. 3 is the node around 60 *a*_0_ and the absence of appreciable probability in the two asymptotically closed channels for internuclear separations larger than 50 *a*_0_. In the rest of this paper we adopt the convention of calling this node the last node in the wavefunction, even though the wavefunction keeps oscillating with a wavelength corresponding to a kinetic energy of 500 μK. The *E* = 0 wavefunction will always have a last node associated with the number of bound states in the potential (see Appendix A), and this nodal position does not change significantly for wavefunctions with kinetic energies below 1 mK. A more general expression for the asymptotic wavefunction in Eq. (4) replaces sin(*k*(*R* − *a*_1,−1_)) with sin(*kR* + *δ*(*k*)) where the phase shift *d* has as a limiting behaviour − *a*_1,−1_*k* for small collision energies. The answer to the question “what is small?” is system-dependent, but for Na the answer is about 1 mK or less. Moreover, for these collision energies and for internuclear separations *R* at which the long-range dispersion potential has died off sufficiently compared to the kinetic energy, the product *kR* is *still* small compared to one and the wavefunction in Eq. (4) can be approximated as being proportional to *k*^1/2^(*R* − *a*_1,−1_). The wavefunction for higher-order plane waves is proportional to *k*^(2ℓ+1)/2^. This analytic variation with *k* defines the Wigner threshold regime [18,19].

**Fig. 4:**
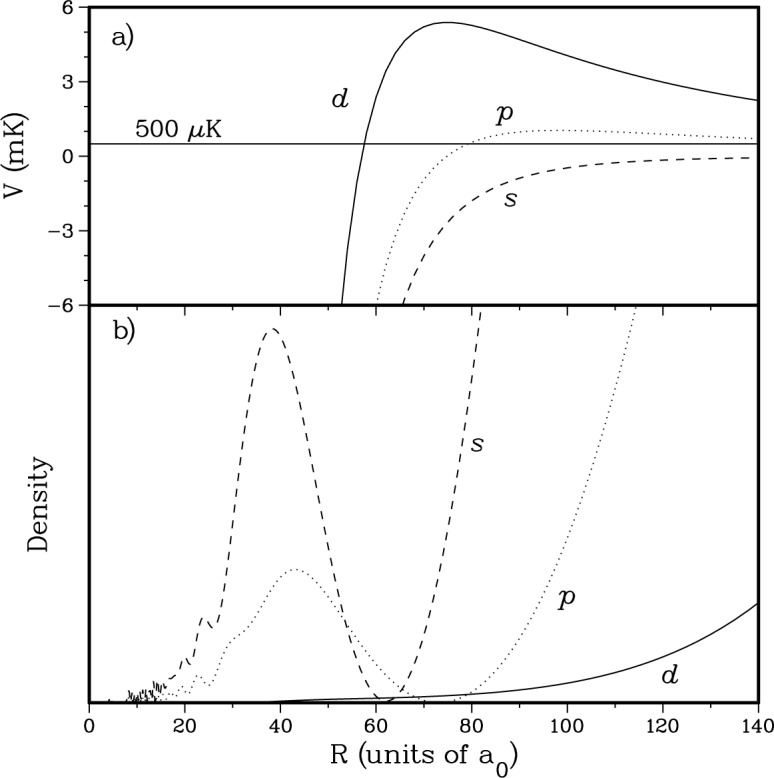
a) The *s*, *p*, and *d*-wave potential barriers as a function of internuclear separation. b) The probability densities for the three wavefunctions, corresponding to a 500 μK collision starting from the *f*_a_ = 1, *f*_b_ = 1 spin channel.

In Fig. 4 we show the radial density of three ground state wavefunctions as a function of internuclear separation. All wavefunctions correspond with a collision starting in a *f*_a_ = 1, *f*_b_ = 1 channel with 500 μK kinetic energy. The density is obtained from the multichannel wavefunction $|\Psi_{Fp\ell ff_{a}f_{b}}^{E\left(+\right)}\rangle$ by summing the squares of the $\phi_{f_{a}f_{b}}^{\left(+\right)}$ (*R*) at each *R*. In particular, the graph shows the $\Psi_{s2}^{\left(+\right)}$, $\Psi_{p1}^{\left(+\right)}$, and $\Psi_{d2}^{\left(+\right)}$ waves. Moreover, Fig. 4 shows the *s*-, *p*-, and *d*-wave potentials of the *f*_a_ = 1, *f*_b_ = 1 component of the potential matrix. In the radial region that is important for the photoassociation spectroscopy of the $0_{g}^{-}$ state this diagonal element of the multichannel potential matrix is given by − *C*_6_/*R*^6^ + (*ℏ*^2^/2*μ*)ℓ(ℓ + 1)/*R*^2^. This is a consequence of the fact that for these internuclear separations the two ABO potentials are identical and given by their dispersion form. Moreover, the density for *R* > 50 *a*_0_ is solely due to *f*_a_ = 1, *f*_b_ = 1 component of the wavefunction.

For Na the height of the *d*-wave barrier maximum at 75 *a*_0_ is 5.4 mK. This is much higher than the temperature (~ 500 μK) of the atoms in the MOT. Therefore, the penetration of the *d*-wave into the region near 75 *a*_0_ is greatly reduced by the centrifugal barrier. In fact, full close-coupled calculations show that, for Na MOT temperatures, ℓ > 1-wave wavefunctions outside of the barrier are almost independent of the shape of the electro-static potentials inside the centrifugal barrier. Therefore, the *d*-wave wavefunction is mainly determined by the well-known long-range form of the potential while higher partial waves do not contribute significantly to the lineshapes. As a result, we find that $\Psi_{d2}^{\left(+\right)}$ and also $\Psi_{d0}^{\left(+\right)}$ are almost identical to a pure *j*_2_(*kR*) spherical Bessel function in the region where the Franck-Condon factors are nonzero (i.e., in the region of the centrifugal barrier) with their normalization determined by asymptotic boundary conditions. This implies that we will have no freedom in modifying the *d*-wave features of the spectra. There is much more penetration of the *s*- and *p*-wave wavefunctions to small internuclear separations and therefore they will display a significant dependence on the shape of the inner wall of the two ABO potentials.

The above is in contrast to the case of ^87^Rb where a *d*-wave shape resonance dominates the spectrum obtained from samples of doubly polarized atoms [28]. In Rb, the *d*-wave barrier is comparable to the most probable collision energy (*k*_B_*T*) and as a result there is significant barrier penetration by the wavefunction. A similar effect could occur in the current Na experiments for the *p*-wave; however, this is in contradiction with the observation of a *p*-wave node near the minimum of the $0_{g}^{-}$ state. Because the *d*-wave barrier height in Na is large compared to the most probable collision energy, any *d*-wave resonance that might occur will be narrow. No experimental evidence exists for such a resonance.

### 3.2 Excited Bound States

The long-range $0_{g}^{-}$ potential results from a spin-orbit avoided crossing between a ^3^Σ_g_ and *a*^3^∏_g_ potential [11–13]. These two non-relativistic electronic curves plus six additional potentials dissociate to the atomic ^2^S + ^2^P asymptote [11]. The notation ^2^*^S^*^+1^*Λσ* reflects the underlying symmetries in the nonrelativistic electronic Hamiltonian, for which the total electron spin *S* is conserved since the electrostatic interactions are independent of spin. The absolute value of the projection of the total electronic orbital angular momentum on the body-fixed symmetry axis (*Λ*) is conserved due to the cylindrical symmetry of the electronic Hamiltonian. The labeling of the molecular states with *σ*, which is either gerade (g) or ungerade (u), is a result of the inversion symmetry of all electrons through the center of mass of the molecule.

Movre and Pichler [11] showed that if one constructs a Hamiltonian based on both electrostatic interactions and the relativistic spin-orbit interaction that results from the *P* atom, then the resulting Hamiltonian mixes electronic states labeled by *SΛΣσ* (where *Σ* is the body-fixed projection of *S*) with states labeled by *S′Λ′Σ′σ*′ such that *Ω* = *Λ* + *Σ* = *Λ*′ + *Σ*′ is conserved and *σ* = *σ*′. In addition, for *Ω* = 0 states the Hamiltonian also separates into two subspaces which have definite symmetry under reflection of the electronic wavefunction through an arbitrary plane containing the internuclear axis. This reflection symmetry is denoted by a superscript + or −. The complete notation for the spin-orbit mixed Hund’s case(c) states is $\Omega_{\sigma }^{\pm }$. The purely long range $0_{g}^{-}$ potential is obtained within this two-state Movre-Pichler model by incorporating only the spin orbit and resonant dipole interactions which are the dominant forces at long range between an alkali ^2^S atom and a ^2^P atom. The two adiabatic $0_{g}^{-}$ potentials are found by diagonalizing the potential matrix:

VMP=Π  Σ(C3R3−2Δ32Δ32Δ3−2C3R3−Δ3) Π Σ, (5)

where *Δ* is the atomic spin-orbit splitting and we have taken the zero of energy to be the ^2^S + ^2^P_3/2_ asymptote. Within this simple model the well depth is *Δ*/9, independent of the resonant dipole interaction strength and the potential minimum is at *R* = (9*C*_3_/2*Δ*)^1/3^. For Na(3 P), *Δ* = 515.520 GHz, *C*_3_ = 4.018 zJ nm^3^ (6.219 a.u.) [14] and *R*_e_ ≈ 72 *a*_0_.

**Fig. 5:**
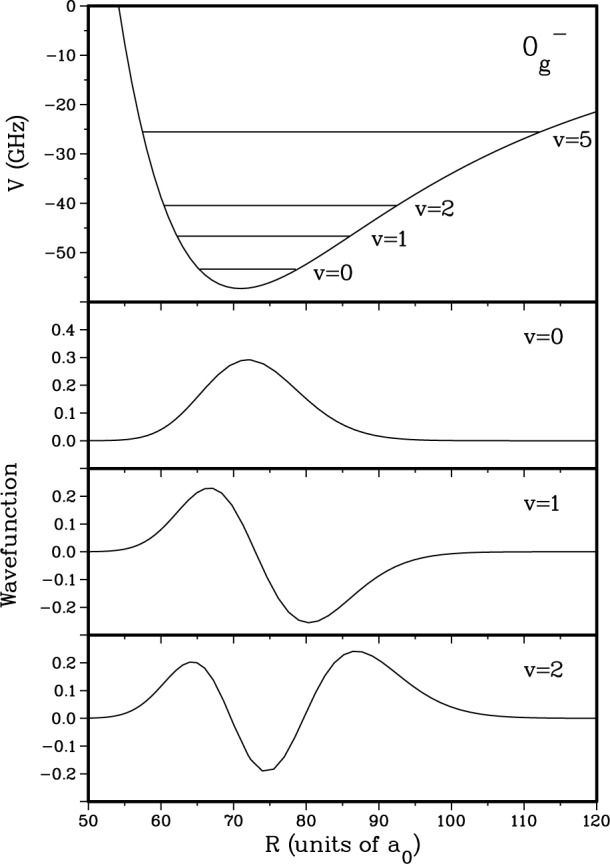
The purely long-range $0_{g}^{-}$ adiabatic potential and selected adiabatic vibrational wavefunctions versus the internuclear separation. The *v* = 0 and 2 wavefunctions are nearly symmetric with respect to the minimum of the well, while the *v* = 1 level is antisymmetric.

Figure 5 shows the purely long range adiabatic $0_{g}^{-}$ potential along with the three lowest adiabatic vibrational wavefunctions in this potential. This is a purely long range potential in the sense that the electron clouds of the two atoms do not overlap in the vicinity of the potential well and it is therefore completely determined by atomic parameters. In the region where these wavefunctions are nonzero, the $0_{g}^{-}$ potential is nearly a harmonic potential and hence, the *v* = 0 and 2 wavefunctions are nearly symmetric with respect to *R*_e_ while *v* = 1 is antisymmetric.

In Ref. [13], three of the present authors discussed the rotational and hyperfine structure of the $0_{g}^{-}$ vibrational levels. There, we showed that we could obtain the exact bound states of the fully rotating 3^2^S + 3^2^P Hamiltonian including hyperfine structure. For a given total angular momentum *F*′ and parity *p*′, the full Hamiltonian matrix will include up to 96 coupled-spin basis states. Although the multichannel wavefunctions in principle can be distributed over as many as 96 spin channels, an appropriate transformation can usually be found that will constrain the nonzero amplitude to at most a few channels. Moreover, the nonzero components of such a wavefunction have a common radial dependence, as depicted in Fig. 5. In other words, the $0_{g}^{-}$ levels for *v* < 9 are essentially adiabatic [13] and thus can be viewed as single-channel wavefunctions. Note, that the actual spin structure is essential for calculating the transition matrix elements which are labeled by *F′p′β*.

For the purely long range $0_{g}^{-}$ states, it turns out that the hyperfine and Coriolis interactions are absent in first order. Therefore, in addition to the quantum numbers *F*′ and *p*′, the quantity *J*′ = *F*′ − *I* is approximately good. Moreover, *J*′ = *S* + *L* + ℓ, where the electron orbital angular momentum *L* = 1. General symmetry relations show that *p*′ = (− 1)^ℓ+1^ for homonuclear ^2^S + ^2^P molecules, i.e., odd ℓ corresponds to even parity and vice versa, and (− 1)^ℓ+^*^s^*^+^*^I^* = − 1 where σ = 0(1) for gerade (ungerade) states. For the $0_{g}^{-}$ states where *S* = *L* = 1 additional selection rules are appropriate. We find *J*′ + *I* is odd or, equivalently, even *J*′ correspond with odd parity and vice versa. Some of these selection rules slowly break down with increasing vibrational quantum number as second order coupling to nearby states with different $\Omega_{\sigma }^{\pm }$ symmetry becomes stronger.

This description of the $0_{g}^{-}$ vibrational levels leads to the following picture of the level structure. The energy level distribution is in first order given by a rotational progression in *J*′. Each *J*′ consists of a group of nearly degenerate levels. The *J*′ = 0 level is two-fold degenerate with *I* = 1 or 3, while the *J*′ = 1, 2, 3, and 4 levels are 4, 8, 6, and 10-fold degenerate, respectively. From Ref. [13] we know that for the lowest three vibrational levels the hyperfine degeneracy is lifted by no more than 5 MHz, which is still small compared with the natural width and the rotational constant.

Even though *J*′ is a good approximate quantum number and behaves as an effective rotation, this does not imply that states with a definite value of the mechanical rotation are formed. In fact, even *J*′’s represent positive parity states and therefore contain even partial waves and odd *J*′’s contain odd partial waves. For example a *J*′ = 2 state will have ℓ = 0, 2, and 4 contributions. The low temperatures in the present experiments limit ℓ to values of 2 or less.

### 3.3 Molecular Rabi Matrix

The molecular Rabi matrix elements $\Omega_{Fp\ell f\alpha }^{F\prime p\prime \beta }$ are obtained by first considering the allowed optical excitation of a pair of atoms by a single photon at large internuclear separation. The Rabi matrix in the atomic hyperfine basis is then transformed into the molecular basis. The basic approach is an extension of that originally used in Ref. [21] where we have incorporated the atomic hyperfine structure. In simple terms, we know the atomic transition dipole moment and the atomic hyperfine selection rules for optical transitions, which are **Δ***f*_a_ = {0, ± 1}, **Δ***l*_a_ = 1, **Δ***s*_a_ = 0, and **Δ***i*_a_ = 0, where we have assumed that the atom labeled “a” has been excited. These selection rules insure that only the orbital angular momentum *l*_a_ changes for the optically dipole allowed ^2^S → ^2^ P transition.

At large internuclear separation we can define a set of atomic scattering states

$$|\ell \mu ,c_{a}f_{a}m_{f_{a}},c_{b}f_{b}m_{f_{b}}\rangle =Y_{\ell \mu }|c_{a}f_{a}m_{f_{a}}\rangle |c_{b}f_{b}m_{f_{b}}\rangle$$ (6)

which are products of magnetically resolved atomic hyperfine states $|c_{\alpha }f_{\alpha }m_{f_{\alpha }}\rangle$for atoms *α* = {*a*,*b*} and a spherical harmonic wavefunction *Y*_ℓ_*_m_* which describes the mechanical rotation of the two atoms about their center-of-mass. In the above description *c_α_* stands for all other quantum labels needed to uniquely specify the atomic hyperfine state—i.e., for a ground state Na atom *c_α_* = 3^2^S while for the first excited state of Na *c_α_* = 3^2^P.

Beginning with an initial set of atomic scattering states $|\ell \mu ,c_{a}=3^{2}S\;f_{a}m_{f_{a}}$, $c_{b}=3^{2}S\;f_{b}m_{f_{b}}\rangle$ and a second set of atomic scattering states $|\ell \prime \mu \prime ,c_{a}^{\prime }=3^{2}P\;f_{a}^{\prime }m_{f_{a}}^{\prime },c_{b}^{\prime }=3^{2}S\;f_{b}^{\prime }m_{f_{b}}^{\prime }\rangle$, where we arbitrarily assume that atom “a” is excited, then it is obvious that we can derive the Rabi matrix elements between these two states from the known atomic transition dipole. In such a picture the Rabi matrix element will be zero unless $\delta_{\ell ,\ell \prime }\delta_{c_{b},c_{b}^{\prime }}\delta_{f_{b},f_{b}^{\prime }}\delta_{m_{f_{b}},m\prime_{f_{b}}}=1$, and the hyperfine selection rules for the optically excited *a*-atom are obeyed. These selection rules insure that only one atom absorbs the photon when the two atoms are at infinite internuclear separation. The real situation is slightly more complicated since we must symmetrize the asymptotic basis with respect to exchange of the identical nuclei.

Our asymptotic derivation of the molecular Rabi matrix is strictly valid for the purely long range $0_{g}^{-}$ state, since the electronic clouds of the two atoms never overlap and distort the atomic dipoles. As a check on the transition dipole moment and a confirmation of our code we can calculate the natural lifetime of an arbitrary molecular state; e.g., the $A^{1}\sum_{u}^{+}$ state or the purely long range $0_{g}^{-}$ state. This involves summing over all ground state hyperfine components and, as expected, yields ~20 MHz for the purely long range $0_{g}^{-}$ state and ~10 MHz for the $A^{1}\sum_{u}^{+}$ state.

### 3.4 Evaluation of the Molecular Transition Strength

The absorption of a photon excites the colliding atoms from a ground state scattering wave into a bound excited state molecule. Although our analysis is based on exact numerical calculations of the molecular Rabi matrix and the ground and excited state multichannel quantum wavefunctions, much physical insight for interpreting our result can be obtained from considering the molecular transition strength labeled by the approximately good quantum numbers discussed above: *J*′, ℓ, and *f*. This transition strength is determined from the Franck-Condon overlap matrix elements:

$$F_{\ell f}^{vJ\prime }\left(E\right)=\sum_{\alpha \beta F\prime F}|\langle \phi_{F\prime p\prime \beta }^{vJ\prime }|\hbar \Omega_{Fp\ell f\alpha }^{F\prime p\prime \beta }|\Psi_{Fp\ell f\alpha }^{E\left(+\right)}\rangle {|}^{2}.$$ (7)

The sum over *α* only involves channels where the two atoms have *f*_a_ = *f*_b_ = 1. The summations over *p* and *p*′ are absent as ℓ uniquely defines the parity of the ground state and *p*′ = − *p* from the selection rules of the transition dipole moment.

The discussion in Sec. 3.1, when combined with the above equation, shows that there are only two possible *s*-wave contributions, corresponding to *f* = *F* = 0 and *f* = *F* = 2. These are designated as $F_{s0}^{vJ\prime }\left(E\right)$ and $F_{s2}^{vJ\prime }\left(E\right)$ respectively. For the purely long range $0_{g}^{-}$ state, the *s*-waves contributes predominantly to the *J*′ = 2 and to a lesser extent to the *J*′ = 0 feature. For the *p*-wave there is only one possible contribution, $F_{p1}^{vJ\prime }\left(E\right)$. The *p*-wave contributes to *J*′ = 1 and 3 features only. Finally, there are two possible *d*-wave contributions, $F_{d0}^{vJ\prime }\left(E\right)$and $F_{d2}^{vJ\prime }\left(E\right)$. The *d*-waves contribute to the *J*′ = 0, 2, and 4 features.

One important aspect of our argument below is that $F_{s2}^{vJ\prime }\left(E\right)\gg F_{s0}^{vJ\prime }\left(E\right)$. Therefore, the analysis of the lineshapes is primarily sensitive to the *f* = 2 *s*-wave and not the *f* = 0 one. One reason for this is that the phase space factor 2*F*′ + 1 is much larger for the *f* = 2 *s*-wave. However, there is no reason why the scattering length *a_f_*_=0_ should be the same as the scattering length *a_f_*_=2_, since the different *f* values lead to slightly different Hamiltonians. Both of these scattering lengths are different from those for the electrostatic potentials for the ${\;}^{1}\sum_{g}^{+}$ and ${\;}^{3}\sum_{u}^{+}$ states without hyperfine structure, because of the strong mixing of these states in the *s*-wave collision for a given *f*. Our complete close coupling calculations show: 1) that *a_f_*_=0_ is actually near *a_f_*_=2_, crossing it as the inner ABO potentials are varied, and 2) that $F_{s2}^{vJ\prime }\left(E\right)\gg F_{s0}^{vJ\prime }\left(E\right)$ is valid for the transitions we study.

Finally, we make a more quantitative argument that near *R*_e_ the harmonic nature of the $0_{g}^{-}$ potential for *v* = 0 − 2 (Fig. 5) helps explain the relative intensities of the *p*-wave features for these levels. Consider the following one-dimensional spinless Franck-Condon factor:

$${\left|\int_{0}^{\infty }dR\phi_{v}\left(R\right)\Psi_{p1}^{\left(+\right)}\left(R\right)\right|}^{2}.$$ (8)

In this equation, *ϕ_v_*(*R*) is the adiabatic $0_{g}^{-}$ vibrational wavefunction and, as discussed above, $\Psi_{p1}^{\left(+\right)}$ is the single *p*-wave for *f*_a_ = 1 + *f*_b_ = 1 collisions. We neglect any *R*-variation in the Rabi matrix elements for different hyperfine components of the upper level. The *v* = 0 function, and to a lesser extent the *v* = 2 function, is nearly symmetric about the minimum near *R*_e_ = 72 *a*_0_, whereas the *v* = 1 function is antisymmetric. Since the *p*-wave has a node so close to *R*_e_, it also is nearly antisymmetric about *R*_e_. Therefore, the molecular transition strength for *p*-waves is very small for *v* = 0 and 2, but much larger for *v* = 1.

## 4. Obtaining the Scattering Length

Having developed these theoretical tools, we now return to the interpretation of the experimental spectra in terms of the *s, p*, and *d* wavefunctions. As explained in Sec. 3, there are three theoretical elements which are needed in order to simulate the experimental spectrum using Eq. (1). These are the ground state wavefunctions $|\Psi_{Fp\ell f\alpha }^{E\left(+\right)}\rangle$, the excited state wavefunctions $|\phi_{F\prime p\prime \beta }^{vJ\prime }\rangle$, and the molecular Rabi matrix elements $\Omega_{Fp\ell f\alpha }^{F\prime p\prime \beta }$. Because of the checks on the transition dipole moment described in Sec. 3.3 we can be confident in the determination of the latter. Refs. [13,14] on the rovibrational-hyperfine states of the Na_2_ $0_{g}^{-}$ state provide compelling evidence that we can calculate the excited states accurately. Thus, the uncertainty in our ability to simulate the experimental spectra is mainly associated with inaccuracies of the $X^{1}\sum_{g}^{+}$ and $a^{3}\sum_{u}^{+}$ RKR potentials, and thus in generating the ground state wavefunctions.

**Fig. 6:**
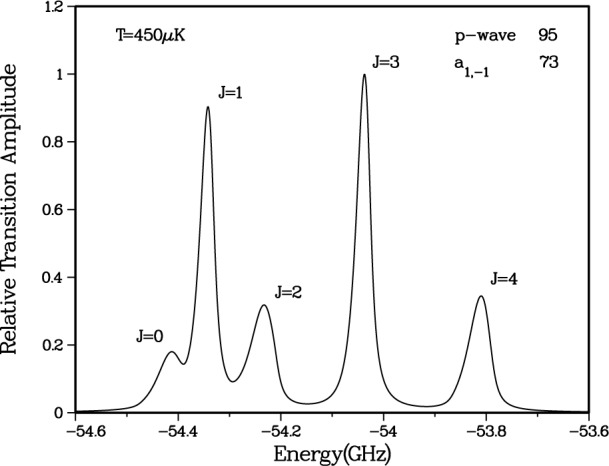
Simulated *v* = 0 spectra for the original RKR potentials. The *J* = 2 peak is completely dominated by the *d*-wave scattering, as can be inferred from its relatively large width of ≈ 60 MHz and the “slow” onset of the red side of the line.

In Fig. 6, we show the *v* = 0 simulated spectrum for our original fit of the ground state Na_2_ RKR potentials [24]. The ground state collision wavefunctions are computed exactly given these $X^{1}\sum_{g}^{+}$ and $a^{3}\sum_{u}^{+}$ potentials. The three elements of the theory are then substituted into Eq. (1) and the thermal lineshape is calculated assuming a temperature *T* = 450 μK. Note that unlike the experimental spectrum (Fig. 2) the simulated spectrum has very large *J* = 1 and 3 peaks and a rather weak *J* = 2 feature. The reason for this is that our fit of the Na $X^{1}\sum_{g}^{+}$ and $a^{3}\sum_{u}^{+}$ RKR potentials caused $\Psi_{s2}^{\left(+\right)}$to have a *a*_1,−1_ scattering length of 73 *a*_0_, with a corresponding *s*-wave node at 78 *a*_0_. This results in a nearly zero Franck-Condon factor for the *s*-wave *J* = 2 feature. For these potentials the *p*-wave node for $\Psi_{p1}^{\left(+\right)}$ was at 95 *a*_0_, far from *R*_e_. This is inconsistent with the experiment and indicates that the RKR potentials must be altered.

**Fig. 7:**
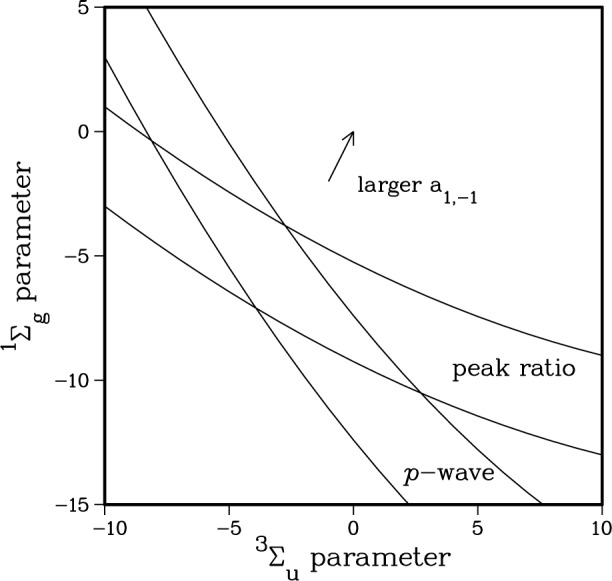
Parameter space plot for variation of $X^{1}\sum_{g}^{+}$ and $a^{3}\sum_{u}^{+}$potentials. Note that smaller values of the parameters imply a deeper potential. The arrow indicates the direction in which the *a*_1,−1_ scattering length increases.

Changing the inner wall of the $X^{1}\sum_{g}^{+}$ and $a^{3}\sum_{u}^{+}$ RKR potentials changes the accumulated phase of the wavefunction or, equivalently, changes the position of the last node. In Fig. 7, we show how varying the inner walls of the potentials modifies various properties which depend on the ground state scattering wavefunction. The two axes represent independent, adjustable parameters which cause a smooth change in the inner wall of the $X^{1}\sum_{g}^{+}$ and $a^{3}\sum_{u}^{+}$ potentials respectively. The precise form of the adjustable parameter is irrelevant [29] since we are only sensitive to the accumulated phase up to the Franck-Condon region (*R* > 50 *a*_0_), where the potentials are completely determined by atomic properties. The plotted lines forming two distinct bands correspond to lines of constant position of the last *p*-wave node and constant ratio of the *J* = 2 and *J* = 4 peak heights. The intersection of the bands in Fig. 7 determines the allowed range of the scattering length.

**Fig. 8:**
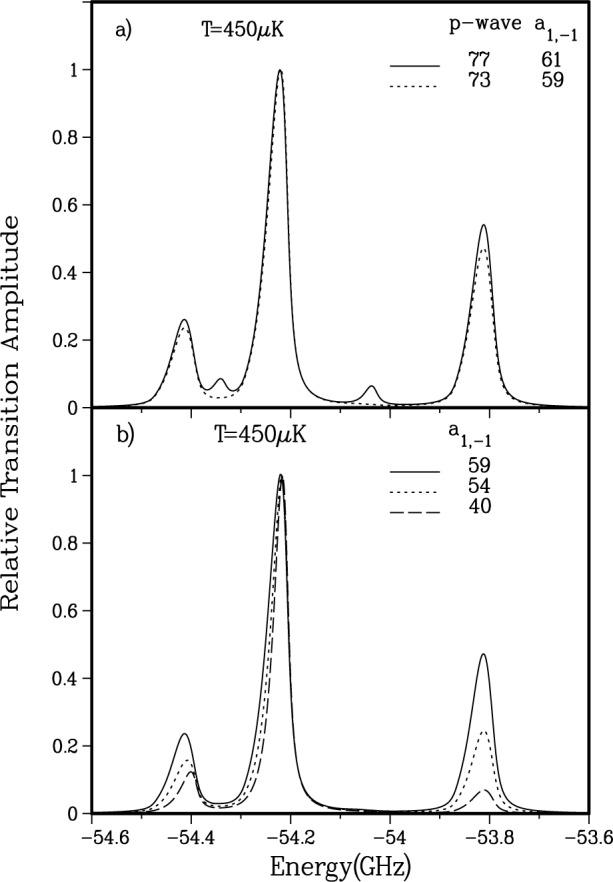
Simulated *v* = 0, $0_{g}^{-}$ spectrum for various potentials. The exact transition dipole moment is used: a) shows the effects of moving the *p*-wave node while *a*_1,−1_ is held nearly constant and b) shows the effects of moving *a*_1,−1_ while the *p*-wave node is fixed at 73 *a*_0_.

Fig. 8a shows how the simulated spectrum changes when the *p*-wave node moves to smaller *R* for nearly constant *a*_1,−1_ scattering length. The spectra have been normalized with respect to the *J*′ = 2 peak. Notice that a relatively small change in the *p*-wave node position has a marked effect on the odd *J*′ peaks in the spectra. Hence to have very weak *v* = 0, *J*′ = 1 and *J*′ = 3 peaks, consistent with the experimental data, we find that $\Psi_{p1}^{\left(+\right)}$ must have a node close to *R*_e_. The calculations strongly constrain the *p*-wave node to 73 *a*_0_ 6 3 *a*_0_. This defines the *p*-wave band in Fig. 7. Note that there is a range of $X^{1}\sum_{g}^{+}$ and $a^{3}\sum_{u}^{+}$ potentials which satisfy this constraint.

In the discussion of the optimal position of the last *p*-wave node we used the wavefunctions with 500 μK kinetic energy in the incoming spin channel. Unlike for *s*-wave scattering, where in the Wigner threshold regime the nodal positions are independent of the collision energy, the position of the *p*-wave node always shifts with collision energy. In fact, the zero energy wavefunction has a node which is about 2 *a*_0_ to 3 *a*_0_ inside the reported *p*-wave node. The 500 μK collision energy is close to the most probable collision energy in a MOT, and therefore the spectra are most sensitive to the position of this node.

Having determined the position of the last *p*-wave node, we now argue that the corresponding *f* = 2 *s*-wave node lies at smaller *R*. This has been confirmed by independent full close coupled calculations, by the theoretical arguments presented in appendix A, as well as being supported the widths of the observed lines. Appendix A also gives an analytical one-to-one correspondence between the *s*-wave nodes and the scattering length. For now it is sufficient to keep in mind that for Na the value of the scattering length is always a few *a*_0_ smaller than the position of the last node.

In Fig. 8b the simulated spectra for several trial ground state potentials are shown, keeping the $\Psi_{p1}^{\left(+\right)}$ *p*-wave node fixed. Once again, the spectra have been normalized with respect to the *J*′ = 2 peak. The figure shows that the *J*′ = 4 to *J*′ = 2 peak ratio varies dramatically with the *a*_1,−1_ scattering length. If this were the sole difference we could not be as confident about our final values since experimentally we have seen as much as a factor of two change in the *J*′ = 4 to *J*′ = 2 peak ratios by varying the frequency of the ionization laser. In the simulations, changing the *a*_1,−1_ scattering length while keeping the *p*-wave node fixed also causes a large change in the width of the *v* = 0 *J*′ = 2 feature. This is because the width is determined from a mixture of *s*-and *d*-wave contributions: an increased *d*-wave contribution implies a larger width. The *J*′ = 2 width decreases with decreasing scattering length because the *d*-wave contribution becomes less and less important as the *s*-wave Franck-Condon factor increases. Thus, the width of the *J* = 2, *v* = 0 feature can also be used in constraining the scattering length.

As explained in Sec. 3.1, the *d*-wave wavefunction is given by a spherical Bessel function, $j_{2}\left(kR\right)/\sqrt{k}\to k^{5/2}R^{2}$ as k→0, independent of the shape of the potential because the centrifugal barrier inhibits penetration of the wavefunction into the region of interest, as seen in Fig. 4. Thus, the intensities of the *d*-wave features in our simulated spectrum are fixed. This has been confirmed computationally for all the various potentials used in this modeling. However, changing the *s*-wave node and thereby the *a*_1,−1_ scattering length changes the amplitude of the *s*-wave scattering wavefunction in the vicinity of the minimum of the $0_{g}^{-}$ potential, and thus the strength of the *s*-wave features. Moreover, as the *s*-wave character of the *v* = 0, *J*′ = 2 peak increases, the linewidth of the feature becomes narrower. Therefore if the *s*-wave node lies too far from *R*_e_ the *J*′ = 2 feature becomes larger and narrower, as is seen clearly in Fig. 8b. A comparison with the experimental width of the *J*′ = 2, *v* = 0 peak leads us to conclude that a considerable *d*-wave contribution is present and thus the *s*-wave node cannot lie to far from *R*_e_. This reasoning, however, does not tell us on which side of *R*_e_ the *s*-wave node is situated.

We can use the spectra of the higher vibrational levels to further constrain the position of the *s*-wave. The ratio of the purely *d*-wave *J*′ = 4 peak to the *s*-wave component of the *J*′ = 2 peak is proportional to the square of the ratio of the ground state wavefunctions at a characteristic distance *R_v_* [30]. A simple estimate of the intensity ratio of the *s*-wave and *d*-wave contributions to the spectral lines can be made based on the approximate wavefunctions for the *s*- and *d*-waves and is given by:

$$\frac{d}{s}\sim {\left(\frac{k^{5/2}R_{v}^{2}}{k^{1/2}\left(R_{v}-a\right)}\right)}^{2}=\frac{k^{4}R_{v}^{4}}{{\left(R_{v}-a\right)}^{2}},$$ (9)

where we use the *k* → 0 expression of $j_{2}\left(kR_{v}\right)/\sqrt{k}$ for the *d*-wave and $j_{0}\left(k\left(R_{v}-a\right)\right)/\sqrt{k}$ for the *s*-wave, and *a* is the scattering length. We can conveniently take *R_v_* to be the outer turning point of the $0_{g}^{-}$ *v* level. An improvement of the model of the peak ratios involves replacing *a* with the position of the last *s*-wave node. This follows from the modification of the *s*-wave wavefunction due to the long range − *C*_6_/*R*^6^ potential and is discussed in Appendix A. The *k* dependence shows that, as expected, the *J*′ = 4 peaks will disappear for lower temperatures.

The *J*′ = 2 peak is the dominant feature in the experimental spectra of the *v* ≤ 12 vibrational levels. The outer turning points of these levels are between 70 *a*_0_ and 200 *a*_0_. By Eq. (9) an *s*-wave node at these internuclear separations would imply a much stronger *J*′ = 4 peak relative to the *J*′ = 2 peak than observed. We thus conclude that there is no *s*-wave node between 70 *a*_0_ and 200 *a*_0_. Since we have already shown that a node too far away from *R*_e_ leads to an unacceptably small *d*-wave contribution to the *v* = 0 spectrum, we can also immediately rule out a node larger than 200 *a*_0_. Furthermore, a small value for the location of the node is also unacceptable as it leads to a *d*-wave feature that is unacceptably weak and a *v* = 0, *J*′ = 2 level that is unacceptable narrow. Numerical calculations of the peak ratio as a function of the shape of the potentials confirm these simple arguments.

**Fig. 9:**
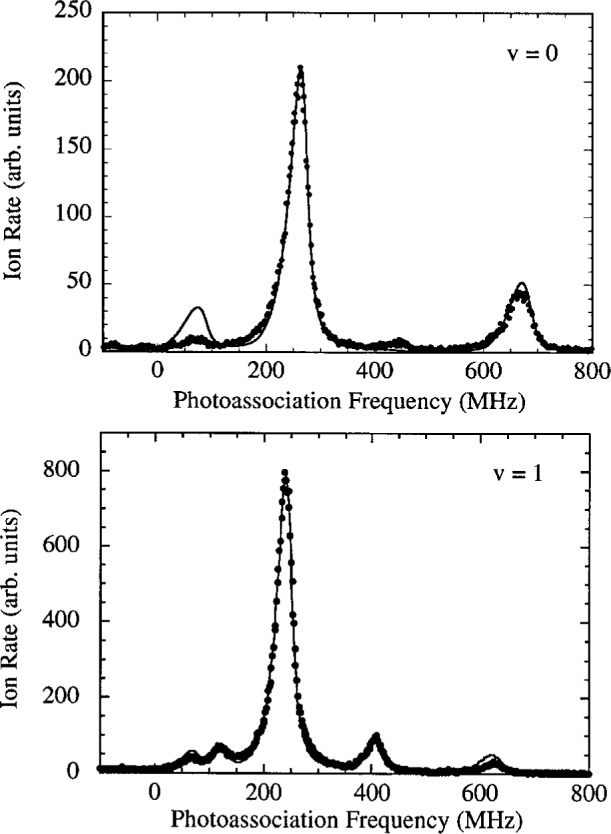
Comparison of theoretical and experimental rotational spectra for *v* = 0 and *v* = 1. The theory is scaled to agree with the experimental *J*′ = 2 peak height and shifted slightly (~2 MHz) in frequency.

Plotting the *J*′ = 2 to *J*′ = 4 peak ratio as a function of the shape of the potentials gives the band labeled “peak ratio” in Fig. 7. The shape of the potentials at which the two bands intersect is the optimal form. Fig. 9 compares the theoretical spectra calculated using the best ground state potentials with the experiment. The only adjustable parameters are the overall height, which is adjusted to fit the observed *J*′ = 2 peak and the absolute frequency which is adjusted by ~ 2 MHz. The relative peak positions and heights are determined from the theory.

From our final potentials we find *z*_0_ = 60 *a*_0_ ± 3 *a*_0_, *z*_1_ = 73 *a*_0_ ± 3 *a*_0_ for the positions of the last *s*- and *p*-wave nodes, respectively and *a*_1,−1_ = 52 *a*_0_ ± 5 *a*_0_. Quoted uncertainties are one estimated standard deviation (combined standard uncertainty). Other scattering properties can be evaluated as well. For example, the scattering length *a*_2,2_ of two atoms with *f_α_* = 2, *m_f_* = 2, is 85 *a*_0_ ± 3 *a*_0_. This is the scattering length relevant in experiments aiming at Bose condensation in doubly polarized samples of Na atoms.

The Na *a*_1,−1_ scattering length has been discussed in the literature before. An experimental measurement of *a*_1, −1_ = 92 *a*_0_ ± 25 *a*_0_ [31] was based on the thermalization time of a sample with a temperature of 200 μK. A theoretical treatment based on improving on the semi-classical RKR potentials with an inverted perturbation approach obtained $86_{-23}^{+66}\;a_{0}$ (Ref. [26]). These values are consistently larger than our value, although in agreement within two sigma if the uncertainties are taken to be one sigma. Even without our detailed numerical calculations, the observed spectra show that the last *f* = 2, *s*-wave node cannot lie between 70 *a*_0_ and 200 *a*_0_.

## 5. Conclusion

An analysis of the rotational lineshapes in photoassociation spectra of the purely long-range Na_2_ $0_{g}^{-}$ state, particularly the lowest vibrational level, places constraints on the possible positions of nodes in the 3^2^S(*f* = 1) + 3^2^S(*f* = 1) scattering wavefunctions. By combining this information with the known spectroscopy of the Na_2_ ground states we generate a set of potentials which produce scattering phase shifts consistent with our observed spectra. From the potentials we calculate the *s*-wave scattering lengths needed as input for theories describing Bose condensates.

Our results reported here are preliminary in that they are based on a small data set which limits our ability to quantify the effects of the ionizing laser. In future experiments we plan to acquire a larger data set and also investigate spectra in which one or both of the colliding atoms are in the 3^2^S(*f* = 2) state. We predict that these spectra will be dramatically different from the ones reported here and their observation will provide an important cross check on the potentials we have derived.

## Figures and Tables

**Fig. 10:**
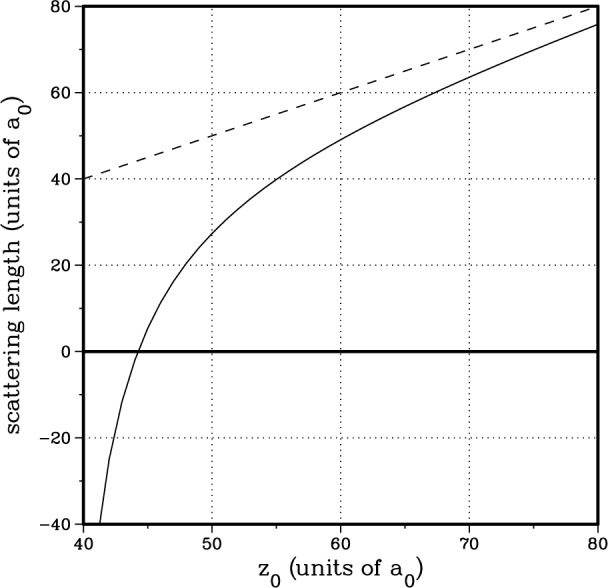
The scattering length versus the last node *z*_0_ in the zero-energy scattering wavefunction. The figure shows the scattering length for two assumptions regarding the long-range behaviour of the potential. The dashed line corresponds to a zero potential for *R* > *z*_0_ and the full line corresponds to a – *C*_6_/*R*^6^ potential for *R* > *z*_0_. The two parameters in the model are the *C*_6_ coefficient and the atomic Na mass.

## 6. Appendix A. From Nodes to a Scattering Length

This Appendix aims to give an intuitive understanding of why for Na_2_ the last *f* = 1, *p*-wave node *z*_1_ of the zero energy wavefunction lies outside the corresponding node of the *f* = 2 or *f* = 0, *s*-wave. We also relate the *s*-wave node to a scattering length.

If we ignore the hyperfine contribution in the multi-channel *f* = 1 and *f* = 2 Hamiltonians the sole difference between the two Hamiltonians is the centrifugal barrier ℓ(ℓ + 1)/2*mR*^2^ where ℓ = 1 or 0, respectively. Decreasing ℓ from one to zero in a continuous fashion makes the interaction slightly more attractive and, hence, increases the phase that the zero energy wavefunction accumulates when integrating from *R* = 0, where the wavefunction is zero, to the position of the last *p*-wave node. Therefore, an *s*-wave node lies just inside *z*_1_. However this does not prove that it is the last *s*-wave node. The wavefunction could accumulate enough phase in the larger *R* region that it has one more node, i.e., the *s*-wave potential could have one more bound level than the *p*-wave potential. The Na hyperfine interaction adds small corrections to this picture. This nodal pattern is confirmed by full multi-channel close coupling calculations for a variety of realistic $X^{1}\sum_{g}^{+}$ and $a^{3}\sum_{u}^{+}$ potentials. For heavier alkali-metal atoms, however, such a conclusion need not apply as the hyperfine interaction is larger and the centrifugal barrier much lower. We will assume that *z*_0_ stands for the node in $\Psi_{s2}^{\left(+\right)}\left(R\right)$. The node of the *f* = 0 *s*-wave wavefunction, $\Psi_{s0}^{\left(+\right)}\left(R\right)$, is closely related to *z*_0_.

From Sec. 3.3 we know that the wavefunction for *R* > 30 *a*_0_ is described in terms of a single potential and the exact wavefunction has a node in this region. This allows us to ignore multi-channel complications. The connection between the last node and the scattering length for the collision between two *f*_a_ = 1, $m_{f_{a}}=-1$atoms is, therefore, far more tractable. If the atom-atom interaction were zero beyond the position of this zero the connection is trivial with *a*_1,−1_ = *z*_0_. The attractive long-range dispersion interaction however is still important. In first order the correction to the scattering length due to the van der Waals interaction has the form [17,32]

$$\begin{array}{c} a_{1,-1}-z_{0}\approx \underset{k\to 0}{\lim}\frac{2\mu }{\hbar^{2}k^{2}}\\ \int_{z_{0}}^{\infty }dR\sin\left(k\left(R-z_{0}\right)\right)\left\{-\frac{C_{6}}{R^{6}}\right\}\sin\left(k\left(R-z_{0}\right)\right) \end{array}$$ (10)

$$=-\frac{2\mu C_{6}}{\hbar^{2}}\int_{z_{0}}^{\infty }dR\frac{{\left(R-z_{0}\right)}^{2}}{R^{6}}=-\frac{2\mu C_{6}}{\hbar^{2}}\frac{1}{30z_{0}^{3}}<0.$$ (11)

For example, if we take the view that *z*_0_ = *z*_1_ ≈ 70 *a*_0_ Eq. (10) implies *a*_1,−1_ = *z*_0_ − 6 = 64 *a*_0_. A more elaborate theory is constructed starting from a zero-energy scattering wavefunction $\psi_{11}^{\left(+\right)}\left(R\right)$ as an asymptotic expansion in 1/*R*. The first terms in this expansion can be shown to be

$$\psi_{11}^{\left(+\right)}\left(R\right)=\left(R-a_{1,-1}\right)+\frac{2\mu }{\hbar^{2}}C_{6}\left\{-\frac{1}{12R^{3}}+\frac{a_{1,-1}}{20R^{4}}\right\}+O\left((\frac{2\mu }{\hbar^{2}}C_{6}{)}^{2}\frac{1}{R^{7}}\right)$$ (12)

where *a*_1,−1_ is the scattering length. This wavefunction must be zero at *z*_0_ leading to

$$a_{1,-1}=z_{0}\frac{1-{\tilde{C}}_{6}/\left(12z_{0}^{4}\right)}{1-{\tilde{C}}_{6}/\left(20z_{0}^{4}\right)}\;\text{with}\;{\tilde{C}}_{6}=2\mu C_{6}/\hbar^{2}.$$ (13)

From this expression it follows that for *z*_0_ ≈ 42 *a*_0_ the scattering length goes to infinity, or equivalently an extra bound level appears. According to Ref. [32] for a pure 1/*R*^6^ potential the exact $\psi_{11}^{\left(+\right)}\left(R\right)$ is known analytically as a linear combination of $\sqrt{r}J_{1/4}\left(x\right)$ and $\sqrt{r}J_{-1/4}\left(x\right)$ with $x=\sqrt{2\mu C_{6}/\hbar^{2}}/\left(2R^{2}\right)$ which leads to a scattering length in terms of a zero of the wavefunction given by

$$a_{1,-1}=\frac{\sqrt[{4}]{{\tilde{C}}_{6}}}{2}\frac{J_{-1/4}\left(x_{0}\right)}{J_{1/4}\left(x_{0}\right)}\frac{\Gamma \left(3/4\right)}{\Gamma \left(5/4\right)}\;\text{with}\;x_{0}=\frac{\sqrt{2\mu C_{6}/\hbar^{2}}}{2z_{0}^{2}},$$ (14)

where *J_n_*(*x*) is the Bessel function. The scattering length as defined in Eq. (14) again has poles, i.e., goes to± ∞, as a function of the position of a node in the zero-energy *s*-wave wavefunction. These poles occur at the zeros of the function *J*_1/4_(*x*_0_). For Na this implies that if the last *s*-wave node is at *z*_0_ = 37.6 *a*_0_ the scattering length is infinite or, alternatively, a bound state at threshold has appeared. For *z*_0_ smaller than this critical value another node much further out appears. In Fig. 10 the scattering length as defined in Eq. (14) as a function of the last node in the zero-energy wavefunction is shown. For *z*_0_ around 70 *a*_0_ to 80 *a*_0_ the effects of the − *C*_6_/*R*^6^ are small and *a*_1,−1_ ≈ *z*_0_. Near *z*_0_ = 45 the scattering length becomes negative and for 37.6 *a*_0_ will become infinitely large.

The long-range potential is not a pure 1/*R*^6^ potential. The *C*_8_ and higher order terms in the polarization interaction must be included. However, they are small for internuclear separations larger than 30 *a*_0_. In fact, the size of the corrections fall inside the 5 % uncertainty of *C*_6_ quoted by Ref. [25] and from Eq. (11) it follows that this adds at most 1 *a*_0_ to 2 *a*_0_ to the final uncertainty in the scattering length.
